# Postoperative Nausea and Vomiting following Endoscopic Sinus Surgery under the Guidance of Adequacy of Anesthesia or Pupillometry with Intravenous Propofol/Remifentanil

**DOI:** 10.3390/ph17010002

**Published:** 2023-12-19

**Authors:** Michał J. Stasiowski, Nikola Zmarzły, Beniamin Oskar Grabarek, Jakub Gąsiorek

**Affiliations:** 1 Chair and Department of Emergency Medicine, Faculty of Medical Sciences in Zabrze, Medical University of Silesia, 41-808 Katowice, Poland; gjakub93@gmail.com; 2 Department of Anaesthesiology and Intensive Care, 5th Regional Hospital, 41-200 Sosnowiec, Poland; 3 Collegium Medicum, WSB University, 41-300 Dabrowa Gornicza, Poland; nikola.zmarzly@gmail.com (N.Z.); bgrabarek7@gmail.com (B.O.G.)

**Keywords:** postoperative nausea and vomiting, pupillary reflex dilatation, total intravenous anesthesia, surgical pleth index, endoscopic sinus surgery

## Abstract

Postoperative nausea and vomiting (PONV) constitutes an adverse event after endoscopic sinus surgery (ESS) under general anesthesia (GA) with intravenous opioids, such as remifentanil (RMF). Monitoring the nociception/antinociception balance using the surgical pleth index (SPI) or pupillary dilatation reflex (PRD) helps guide intravenous RMF infusion. We aimed to investigate whether their employment could help reduce the incidence of PONV in patients undergoing ESS. The data of 30 patients from the GA group, 31 from the SPI group, and 28 from the PRD group were analyzed. The initial RMF infusion rate of 0.25 µg/kg body weight/minute was increased by 50% when the SPI, PRD, or Boezaart Bleeding Scale (BBS) were elevated by >15, >5%, or >2 points, respectively, until they normalized. PONV was present in 7/89 patients (7.9%): 2/31 patients (6.5%) of the SPI group, 1/30 patients (3.3%) of the GA group, and 4/28 patients (14.3%) of the PRD group. Neither PRD nor SPI guidance for RMF administration reduced the incidence of PONV compared to standard practice. Further studies are required in order to investigate the possibility of PONV eradication in patients undergoing ESS under GA when it is possibly combined with paracetamol/metamizole preventive analgesia, as well as those using antiemetic prophylaxis based on the Apfel Score and premedication with midazolam.

## 1. Introduction

Endoscopic sinus surgery (ESS) is a very commonly performed otolaryngological procedure. Postoperative nausea and vomiting (PONV) not only constitutes a challenge for medical staff providing postoperative care after ESS but is also one of the most common unpleasant and distressing adverse events following surgery under general anesthesia (GA), not to mention its impact on early recovery and the cost-effectiveness of the overall hospitalization [1]. Numerous countermeasures are being taken to reduce its incidence, such as opioid-free anesthesia, total intravenous anesthesia (TIVA) [2], pharmacological prophylaxis [3], and non-pharmacological techniques [4]. Since intravenous opioids bear dose-dependent responsibility for the incidence of adverse events, digital guidance of their intraoperative administration may possibly reduce their unwelcome impacts. Various digital intravenous opioid delivery techniques based on nociception/antinociception balance detection, such as antinociception index (ANI), surgical pleth index (SPI), pupillary dilatation index (PRD), and nociception level (NoL), are gaining popularity, opening up new horizons in terms of improving hemodynamic stabilization and reducing the awakening time and the incidence of postoperative complications [5].

The primary objective of the current study was to investigate the advantages of intraoperative analgesia monitoring with SPI- or PRD-guided remifentanil (RMF) administration to manage the volume of total intraoperative blood loss, the state of the surgical field, and the length of the operation compared to standard practice based on the Boezaart Bleeding Scale (BBS) [6]. Currently, as a secondary outcome, we have analyzed the incidence of PONV in patients undergoing ESS in order to investigate the potential impact of monitoring the balance of nociception/antinociception for intraoperative RMF titration during TIVA with propofol on the potential reduction in incidences of the aforementioned postoperative adverse events.

## 2. Results

**Figure A1:**
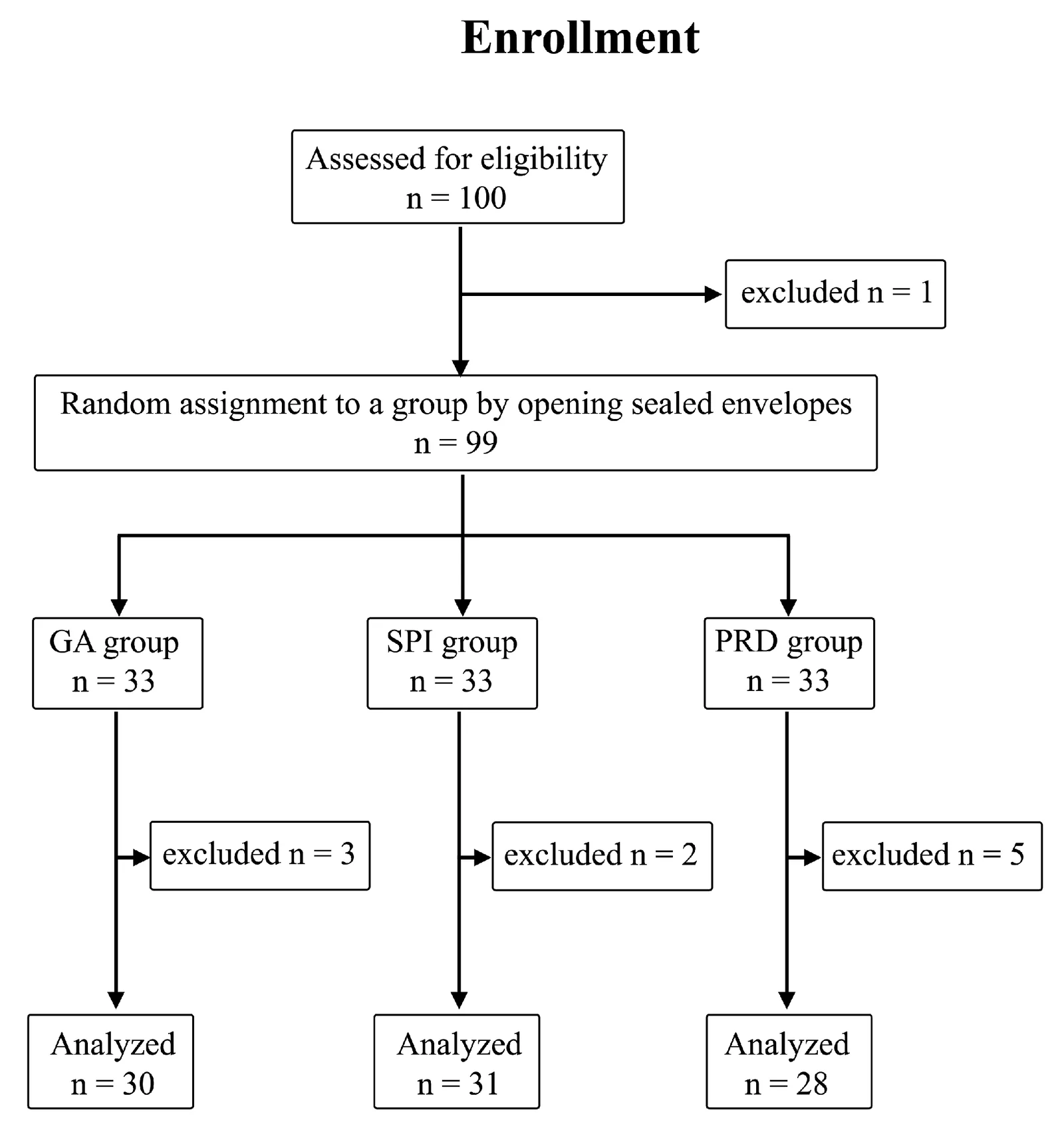
Randomization graph. GA, general anesthesia; SPI, surgical pleth index; PRD, pupillary dilatation reflex.

Out of all 100 patients, one withdrew consent to participate. The remaining patients were randomly assigned to one of three groups by opening sealed envelopes, as presented in Figure A1 [6]. Ten patients were then excluded for anesthesiologic or surgical reasons. Detailed characteristics of the patient’s anthropometric data were previously published [6] (Table A1). There were no significant differences in the analyzed data between the study groups.

Only 7 out of 89 (7.9%) patients declared overall PONV: 1 patient in the GA group (3.3%), 2 patients in the SPI group (6.5%), and 4 patients in the PRD group (14.3%). For further analysis, the incidences of nausea and vomiting were considered separately in PACU and DoL (Table 1).

Regarding the drugs used, significant changes were recorded only in the case of paracetamol. Coincidences in the aforementioned differences are a consequence of the relatively low prevalence of its use in the GA group in the DoL due to individual patients’ needs for additional rescue analgesia. For the remaining variables in the table, the χ^2^ test was not performed due to too-small group sizes or a lack of data.

**Figure 1:**
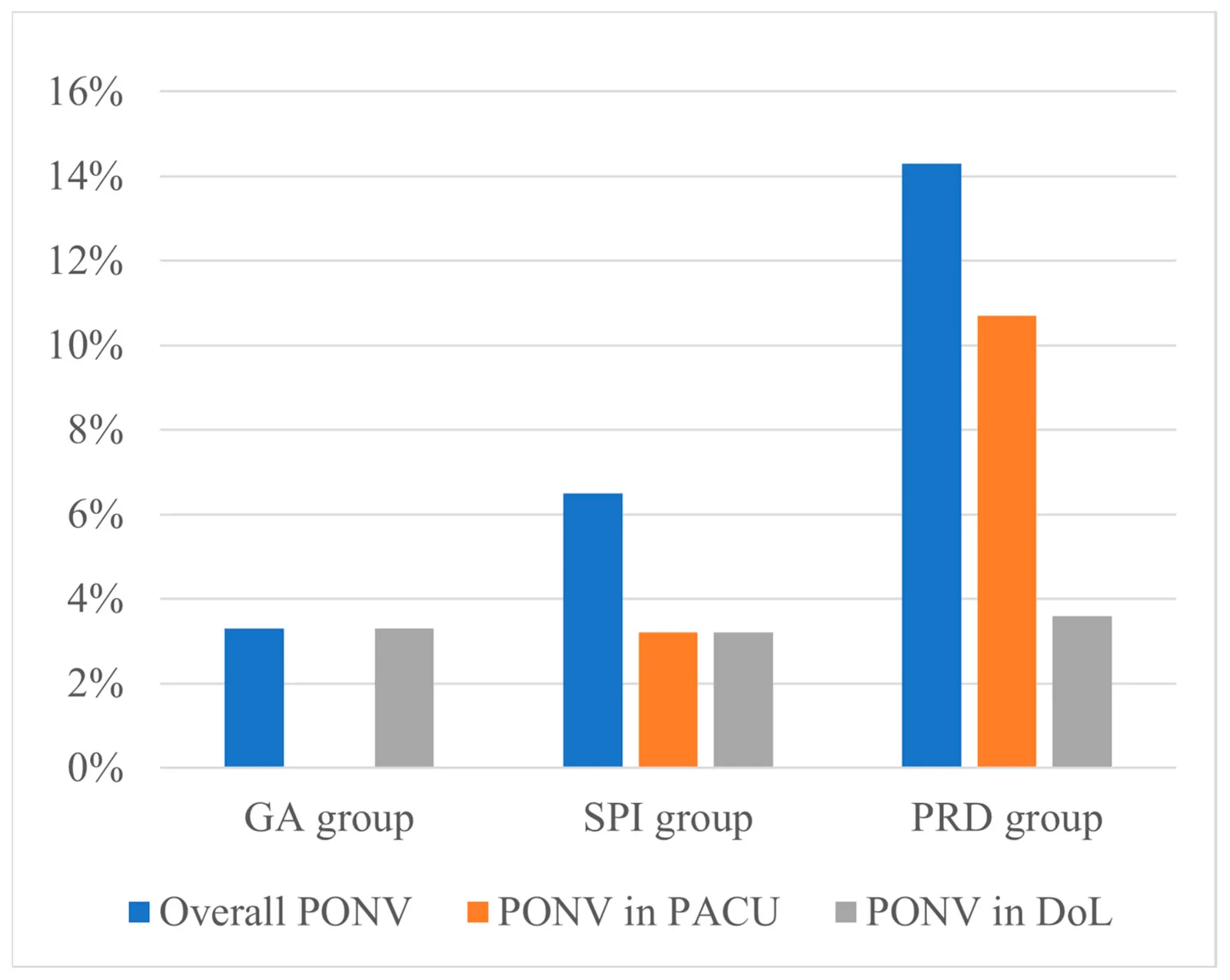
Percentage of general, early, and late PONV. GA, general anesthesia; SPI, surgical pleth index; PRD, pupillary dilatation reflex; PONV, postoperative nausea and vomiting; PACU, Post-Anesthesia Care Unit; DoL, Department of Laryngology.

Figure 1 shows the percentages of overall, early, and late PONV.

Early PONV was not reported in the GA group. In the SPI group, early and late PONV occurred at the same frequency. In the PRD group, the highest percentage was found for early PONV.

Apfel’s overall scale and detailed characteristics are presented in Table 2. There were no significant differences between the study groups in the case of Apfel (%). A lack of statistical significance was also noted for gender and smoking. In the case of motion sickness, history of PONV, and postoperative opioid use, the χ^2^ test was not performed due to the too-small group sizes, similar to Apfel scores of 0 and 3. In turn, the result of the analysis for Apfel scores of 1 and 2 showed no significant differences between the studied groups.

## 3. Discussion

PONV constitutes an adverse event of great concern in patients undergoing different types of endoscopic head and neck surgery [7] and may even be the primary responsibility for unexpected hospital admissions after outpatient endoscopic sinus surgery [8], resulting in decreased satisfaction with the medical services provided.

The incidence of PONV varies after otolaryngological surgery and may even reach up to 74% in the case of middle ear surgery [9]. As an anesthetic modality has been identified as a separate risk factor for PONV in patients undergoing ESS [10], different perioperative management techniques were studied to potentially reduce the risk of PONV following ESS, such as the use of a sphenopalatine ganglion block (SPGB) with a local anesthetic to decrease postoperative pain and PONV by reducing postoperative rescue opioid analgesia [11,12]; the use of pharyngeal packing [13,14,15]; avoidance of nitrous oxide in ventilation gas [14]; administration of magnesium sulfate as an adjuvant [16] or dexmedetomidine as part of intraoperative modality instead of RMF [17,18]; desflurane versus total intravenous anesthesia with propofol [19]; intraoperative cerebral oximetry [20]; or even postoperative use of traditional Chinese medicine [21].

In the current study design, no PONV prophylaxis was administered preoperatively. We aimed to detect whether anesthetic management based on the guidance of RMF infusion using digital techniques to monitor the nociception/antinociception balance may constitute an alternative to the use of pharmacological prophylaxis, which is not free from potential side effects [22,23]. This was similar to the methodology used in one of our previous studies concerning PONV following vitreoretinal surgery [24], where we observed a remarkably low overall incidence of PONV in 9% of cases, with a reduced incidence in groups with preventive analgesia using either paracetamol or metamizole as compared to the other groups.

Regardless of group, in this study, only 7.9% of the patients declared PONV, which may be considered a significant achievement compared to the current literature, which estimates its incidence following ESS to be as high as 36% [25]. It is important to bear in mind that intraoperative analgesia was based on RMF infusion with proven proemetic effects at doses used in all patients [26]. In the study by Zhou et al., PONV occurred in 15.1% of patients in the opioid anesthesia group compared to 7% in the opioid-free group, but at the cost of postoperative sedation, an unwelcome complication following head and neck surgery [27]. Thus, our results are twice as good compared to those of the opioid group in the aforementioned study. Unfortunately, the employment of modern digital techniques for intraoperative analgesia guidance based on nociception/antinociception balance monitoring proved to confer no advantage in terms of PONV incidence reduction.

The SPI guidance for intravenous administration of RMF during TIVA, based on the formula where SPI = 100 − (0.67 × PPGAnorm + 0.33 × HBInorm), collected from finger photoplethysmography signals, did not entail complex or time-consuming preoperative preparations. It has also been proven to be more effective than guidance based on the observation of hemodynamic parameters in response to intraoperative vasoactive reactions following surgical procedures [28]. The increase in SPI values after nociceptive afferent stimulation and their return to baseline levels after RMF infusion were accelerated by 50%, as designed in the current study, which simplified the monitoring of intraoperative titration and increased its reliability. Fluctuations in SPI values in response to nociceptive stimulation have been shown to correlate with the RMF serum concentration [29].

PRD is a robust reflex, and the intraoperative guidance of RMF administration in the current study was based on the measurements of pupillary size and reflexes, with portable infrared pupillometers reflecting the balance between human pupillary responses to RMF and nociceptive stimulation [30]. The pupillary dilation pathway is a three-neuron, sympathetically driven pathway [31]. The first-order neuron begins in the hypothalamus and descends through the midbrain to synapse onto the spinal cord’s ciliospinal center of Budge, found between C8 and T2. The second-order neuron, the preganglionic sympathetic neuron, exits the spinal cord through the ventral roots and ascends through the thorax, near the lung apex and subclavian vessels, onto the superior cervical ganglion. The third-order postganglionic neurons travel along the periarterial carotid plexus through the cavernous sinus. These axons then enter the orbit upon the short and long ciliary nerves (branches of V1, the ophthalmic division of CN V—the trigeminal nerve) to synapse on the dilator pupillae muscle, causing pupillary dilation [32]. Monitoring the quality of intraoperative nociception/antinociception balance using pupillometry is useful in pain management because it allows for the evaluation of the opioid effects and anesthetic titration and may also decrease the severity of acute postoperative pain perception and analgesic consumption within the first 12 h after undergoing major gynecological surgery [33].

In the current study, we observed PONV in four patients in the PRD group and two patients in the SPI group, compared with only one case in the control group, which was not statistically significant. Interestingly, the demand for RMF was statistically significantly lower in the PRD group compared to the SPI group (1.3 ± 1.4 vs. 1.8 ± 0.9 mg; *p* < 0.05), and the duration of surgery was shorter in the PRD group compared to the GA group (63.1 ± 26.7 min vs. 82.6 ± 33.1 min; *p* < 0.05), as we reported in our previously published work [6] (Table A2). This was probably due to the observed tendency, although it was not statistically significant, of a shorter mean time duration of BBS > 2 as compared to the other groups, which might be explained by an improved analgesia regimen. Therefore, we hope to analyze the quality of the nociception/antinociception balance achieved using SPI, PRD, or standard practice based on BBS at certain stages of ESS in a separate report, like in our similar study concerning vitreoretinal surgeries [34]. On the other hand, although there is a proven independent association between the time-weighted average RMF dose during surgery >0.2 μg/kg per minute and increases in the risk of PONV [26], the incidence of PONV was unexpectedly highest in the PRD group in the current study. It is worth noting that in all groups, the initial dose of 0.25 µg/kg body weight/minute was administered despite the lack of statistical significance (see Table 1). Therefore, we assume that further optimization of RMF during ESS may be futile and lead nowhere. Further studies should focus on optimizing a multimodal approach based on preventive analgesia and antiemetic prophylaxis. Nevertheless, it is worth mentioning that in the GA group, the incidence of PONV was 3.3%, with RMF administration based on the BBS scale, which is a considerable achievement compared to the data in the literature.

Our result may be explained by the multimodal approach of administering multimodal pharmacology with proven antiemetic potency. This is similar to the study of Laporta et al. [35], who noted PONV in 8% of patients with anesthetic modality based on preemptive acetaminophen and intraoperative RMF administration.

In the course of the current study, all patients received total intravenous anesthesia based on propofol infusion, the antiemetic potency of which has long been proven [36]. Moreover, in our study, all patients received 2.5 g of metamizole as postoperative pain prophylaxis upon emergence from anesthesia, as it possesses indirect antiemetic properties. In addition, postoperatively, depending on individual needs, patients receive postoperative pain treatment according to contemporary guidelines [37]. It was based on rescue paracetamol in 41.6% of cases. This was similar to the study by Kemppainen et al., which emphasized its high effectiveness in pain treatment after ESS [38], with proven indirect antiemetic properties obtained through the sparring effect of rescue opioid analgesia, which evoked the internal cannabinoid system [39]. Similarly, Lee et al., by analyzing the current literature, concluded that the administration of non-steroidal anti-inflammatory drugs decreases the postoperative risk of nausea following sinonasal surgery [40].

In the current study, for laryngological reasons, 13.5% of patients also postoperatively received dexamethasone with antiemetic potency. This plays a renowned role in antiemetic regimens [41], especially in patients undergoing vitrectomy, where the increase in intraocular pressure following PONV may have devastating consequences [42].

Regarding the analysis of PONV risk factors in the current study, we reported a total of 5 cases of PONV in patients with Apfel scores of 0, 1, or 2 out of 87 patients with no indication for PONV prophylaxis, according to contemporary guidelines [43]. We also found a total of 2 cases of PONV in patients with Apfel scores of 3, with an indication for PONV prophylaxis for high-risk PONV patients according to contemporary guidelines, which we intentionally did not perform. It is, therefore, an open question whether it would be worth administering antiemetic prophylaxis to all 87 patients, which would not be free from possible side effects, like prolongation of the QT interval in healthy volunteers even after a low dose of ondansetron [23], in order to possibly avoid PONV in 5 cases from low-risk groups according to Apfel Score Screening. In view of the fact that these patients also have a right to enjoy pleasant postoperative recovery periods, further studies are required in this field.

For example, Khalil et al. studied the efficacy of reducing the PONV incidence with the employment of antiemetic prophylaxis based on ondansetron and promethazine [9]. In their study, the postoperative incidence of PONV was reduced from 74% in the placebo group to 39% in the promethazine group and to 29% in the ondansetron/promethazine group. Moreover, it was observed that the severity of PONV was statistically significantly lower in the ondansetron/promethazine group compared to the other groups, which supports the thesis regarding the utility of PONV prophylaxis based on antiemetic pharmacology. However, they did not perform stratification of the PONV risk, contrary to our study, which found no statistically significant difference between the study groups in terms of PONV risk based on Apfel Score stratification. It is also worth mentioning that in the current study, premedication with midazolam, which was proven to decrease the rate of PONV, was used as part of the anesthesia regimen in all cases, regardless of group. Therefore, its administration proportionally affected the results. Unfortunately, in the current analysis, the multimodal approach involving the synergic antiemetic potency of drugs used postoperatively was unable to displace antiemetic prophylaxis in high-risk patients. We observed PONV in both high-risk patients, contrary to the observation we found in our study concerning ophthalmic patients, where only one patient with a high risk of PONV complained of PONV out of seven high-risk patients [27].

In conclusion, in the current study, an anesthesia regimen with RMF infusion, regardless of the utility of guidance based on digital monitoring of the nociception/antinociception balance, resulted in a low rate of PONV but did not eradicate it completely. Therefore, further studies are required in order to investigate the potential benefits of an anesthetic regimen based on TIVA. This regimen would involve preemptive analgesia consisting of metamizole with paracetamol alongside PONV prophylaxis, based on metamizole premedication and preoperative stratification, and using the Apfel scale to administer pharmacological prophylaxis to at least, but not only, high-risk PONV patients.

The main limitation of the current analysis is the low number of patients with Apfel scores of at least 3, which would constitute exemplary high-PONV-risk patients. Non-smoking females with either motion sickness or a history of PONV are a rarity in populations of patients undergoing ESS, constituting a rather young, mostly male population. Therefore, we encourage colleagues from larger centers to carry out studies in this field on much larger groups of patients. Secondly, nausea is a subjective phenomenon that may be underreported by patients, who may misinterpret nausea as a general illness after anesthesia. Nausea may also occur in underdiagnosed diabetics when fasting is a confounding factor [44]. The current study also included patients belonging to the third class of the American Society of Anesthesiologists Score. Thirdly, we did not analyze the severity of PONV. Fourthly, as pledgets soaked in topical Xylometazoline, an alpha-agonistic substance possibly interfering with beta-receptors in the sympathetic innervation of the pupillometry system, were administered to the nasal cavities near the area of measurement of PRD, this could have influenced its value because its resorption and venous flow direction in the head could not be predicted. Finally, there exists no consistent algorithm in the current literature for the titration of rescue opioid analgesics based on the observation of fluctuations in SPI values [45]; therefore, as in our previous studies, an intraprocedural increase in SPI of 15 compared to the baseline value, assessed during calibration of the SPI sensor between the airway management and the start of ESS (stage 2), was an indication to adjust the speed of RMF infusion [46,47].

## 4. Materials and Methods

### 4.1. Patients

This study enrolled 100 patients with chronic rhinosinusitis who were scheduled for ESS without septoplasty, with or without polyposis. Patients were aged 18–65 years and had American Society of Anesthesiologists (ASA) scores of I–III. The procedures were performed at the Department of Otolaryngology at Regional Hospital No. 5 in Sosnowiec, Poland.

The exclusion criteria included allergy to hypnotics, pregnancy, pre-existing cardiovascular diseases, platelet count <150,000, pathologies in coagulation tests, taking medications interfering with coagulation, risk of intraoperative hypotension requiring an intensive fluid challenge, and administration of vasoactive drugs that may have interfered with hemodynamic monitoring.

The principal investigator (M.S.) performed the randomization by opening sealed envelopes after every patient gave written informed consent to participate in this study and to undergo GA for ESS. This study was carried out in accordance with the 1964 Helsinki Declaration and was approved by the Local Bioethics Committee at the Medical University of Silesia in Katowice on 24 May 2016 (KNW/0022/KB1/50/16). This study was registered in the Clinical Trials Registry (SilesianMU3 NCT03389763; 9 June 2017).

### 4.2. Anesthesia Technique

Before ESS, patients fasted for 12 h. On the day of surgery, they were premedicated with 3.75–7.5 mg of midazolam (Midanium, Polfa Warszawa, Poland). Immediately prior to surgery, patients were administered an intravenous (IV) solution of Optilite at a dose of 10 mL/kg body weight. Patients were preoxygenated (100% oxygen), and a dose of 2 µg/kg body weight of fentanyl (Fentanyl WZF; Polfa Warszawa SA, Warsaw, Poland), as well as a dose of 2.5 mg/kg body weight of propofol (Propofol 1% MCT/LCT Fresenius Kabi, Bad Homburg, Germany), were administered intravenously. Patients were paralyzed with an intravenous dose of 0.6 mg/kg body weight of rocuronium (Esmeron, Organon, Oss, The Netherlands) after the ciliary reflex disappeared. After 1 min and 20 s, they were intubated with an appropriate endotracheal tube with a cuff size of 7.0–8.5 in the supine position. The CO_2_ was maintained at 35–37 mmHg during the induction of general anesthesia. The lungs were ventilated with a low fresh gas flow (0.7 L/min; 2:1 oxygen to air ratio), and a lung-protective strategy with low tidal volume (6 mL/kg of ideal body weight) was utilized.

Standard procedures for monitoring vital signs were used during induction and the procedure, including non-invasive blood pressure (NIBP) every 5 min, systolic arterial pressure (SAP), diastolic arterial pressure (DAP), mean arterial pressure (MAP), heart rate (HR), electrocardiography (ECG), fraction of inspired oxygen (FiO_2_), arterial oxygen saturation (SpO_2_), and end-tidal carbon dioxide (etCO_2_).

Response and state entropy (RE, SE) were used to assess the depth of anesthesia, while muscle relaxation was monitored using the E-NMT module. Surgical pleth index (SPI) calculations were performed using the Carescape Monitor (B650, GE Healthcare, Helsinki, Finland). PRD measurements were carried out via the NeuroLight Algiscan (version 1.15 A5, Marseille, France).

The patients were anesthetized by total intravenous anesthesia (TIVA). To separate venous access, propofol and remifentanil were administered by continuous intravenous infusion (Remifentanil, Ultiva, GlaxoSmithKline Export Ltd., Middlesex, UK). In the case of train-of-four (TOF), an additional dose of rocuronium was always given. The depth of anesthesia was maintained with propofol (SE of approximately 40), as it affects the response to pain stimuli. Patients were each administered a dose of a non-steroidal anti-inflammatory drug intravenously to avoid postoperative pain, in accordance with the guidelines and the individual condition of the patient [37]. Remifentanil and propofol administrations were stopped, and data recording was discontinued. A dose of 0.02 mg/kg body weight of atropine and a reversal agent (Neostigmine Metil Sulfat, Plantigmin Ampul, Polifarma, Istanbul, Turkey) was administered when TOF > 3 and SE > 88. The patients were then successfully extubated in the operating room.

#### 4.2.1. Stage 1

Upon admission to the operating room, an EEG entropy sensor (RE, SE) was attached to the patient’s forehead, a pulse oximeter was placed on the finger opposite the venous access finger, the NIBP cuff was attached to the left arm, and the first values were recorded.

#### 4.2.2. Stage 2

To reduce venous congestion, patients were placed in the 15° reverse Trendelenburg position. After TIVA induction, infusion of Propofol was started at 100 µg/kg body weight/minute, regardless of the group allocation, as intraoperative awareness with recall constitutes the main disadvantage of this anesthetic regimen [48]. RMF infusion was started at 0.25 µg/kg body weight/minute. Randomization was carried out simultaneously.

#### 4.2.3. Stage 3 Intraoperatively

**Figure A2:**
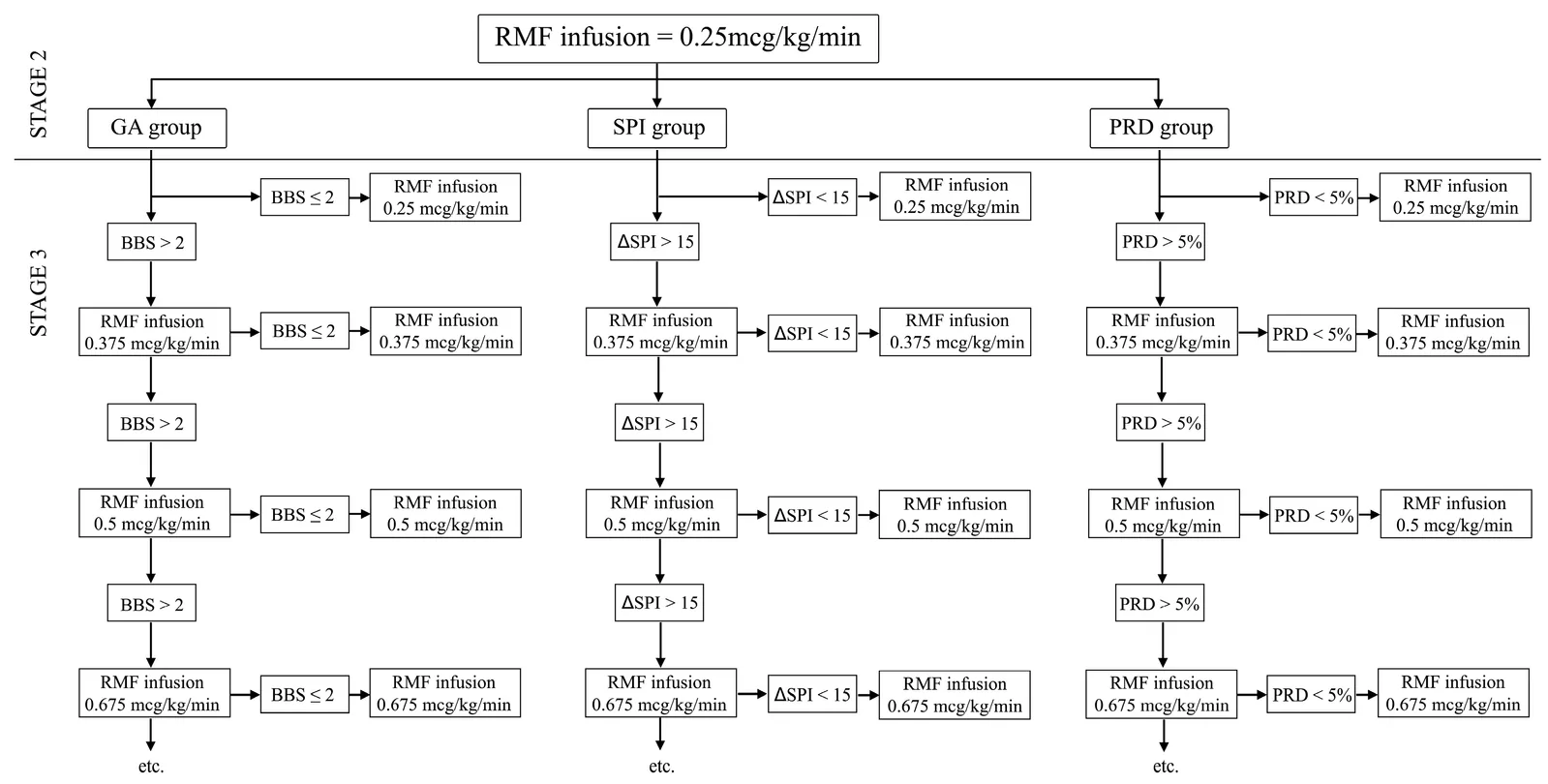
Remifentanil infusion adjustments depend on the group. RMF, remifentanil; GA, general anesthesia; SPI, surgical pleth index; PRD, pupillary dilatation reflex; BBS, Boezaart Bleeding Scale.

Despite the group allocation, the hypnotic component of TIVA was obtained by continuous intravenous administration of propofol. The infusion speed was adjusted to ensure a target SE of around 40, whereas the analgesic component of TIVA was obtained by continuous intravenous administration of RMF. RMF infusion, along with TIVA maintenance, were adjusted by group allocation, as described below and presented in Figure A2 [6].

#### 4.2.4. SPI Group

SPI was measured and recorded every 1 min. When it reached >15 compared to the mean value during stage 2, the RMF infusion rate increased by 50%. An additional fluid challenge (5 mL/kg body weight) was administered when the MAP fell below 65 mmHg.

To avoid hemodynamic complications, when the MAP was <65 mmHg without a response to the fluid challenge, HR < 45/min, BBS < 2, and SPI < baseline, the RMF infusion rate was reduced by 50% and a 10 mg dose of ephedrine (Ephedrinum Hydrochloricum WZF, 25 mg/mL, Polfa Warszawa S.A, Warsaw, Poland) was administered.

#### 4.2.5. PRD Group

PRD was measured and recorded every 15 min. The patient’s eye was flooded with infrared light, and the reflected images were evaluated with an infrared sensor. Differences in pupil size called amplitudes, pupillary light reflex, and pupillary reflex dilatation were assessed and displayed immediately after each measurement with time stamps [49]. Amplitudes were calculated as the percentage of the change in pupillary diameter in millimeters/baseline pupillary diameter and displayed on the screen with a comment (Table 3). Zero sensitivity indicated that the proper RMF infusion speed resulted in a proper nociception/antinociception balance, whereas other sensitivities constituted indications for the acceleration of the RMF infusion speed.

When the PRD was >5%, the RMF infusion rate increased by 50%. An additional fluid challenge (5 mL/kg body weight) was administered when the MAP fell below 65 mmHg. In addition, when the BBS was >2, additional PRD measurements were performed.

Alternatively, when the MAP was <65 mmHg without a response to the fluid challenge, HR < 45/min, BBS < 2, and PRD < 5%, the RMF infusion rate was reduced by 50%, and a 10 mg dose of ephedrine was administered to avoid hemodynamic complications.

#### 4.2.6. GA Group

The Boezaart Bleeding Scale (BBS) values were recorded every 5 min. Table 4 below presents information regarding each grade on the scale [50].

If the value was >2, the RMF infusion rate was increased by 50%. When MAP reached <65 mmHg without a response to the fluid challenge (IV bolus of 5 mL/kg body weight of Optylite solution) or HR was <45/min with a BBS < 2, the RMF infusion rate was reduced to 50% and a 10 mg dose of ephedrine was administered to avoid bradycardia and hypotension.

### 4.3. ESS Technique and Surgical Considerations

After TIVA induction, pledgets soaked in topical vasoconstrictor (Xylometazoline WZF 0.1%, Polfa Warsaw S.A., Warsaw, Poland) were administered to the nasal cavity for the second stage. ESS procedures were carried out by the same otolaryngology specialist with over 10 years of experience. If bleeding occurred and did not respond to topical vasoconstriction, bipolar cauterization was performed, and, if necessary, nasal dressings were applied to the middle meatus. Cerebrospinal fluid was assessed by the operator every 5 min using BBS [51]. The surgery time was counted from the first insertion of the endoscope into the nasal cavity until its final removal.

### 4.4. Postoperative Observation at the Post-Anesthesia Care Unit (PACU) and Department of Laryngology (DoL)

After emergence from TIVA (9–10 points on the Aldrete scale), patients were transferred to the Post-Anesthesia Care Unit (PACU) and observed for at least 1 h. All cases of nausea and vomiting were recorded, and appropriate antiemetic medication was administered intravenously according to the specific needs of the patient until he/she was discharged from the hospital. When unacceptable postoperative pain occurred, a 1 g dose of paracetamol was administered intravenously. In each case of postoperative local edema, a single intravenous dose of 4 milligrams of dexamethasone was administered, depending on the individual needs of the patient.

Since a history of motion sickness or PONV, female gender, not smoking, and postoperative opioid use were considered risk factors, the incidence of PONV was calculated as follows: 10%, 21%, 39%, 61%, and 79% if none, one, two, three, or four of these criteria were met, respectively, according to the Apfel Score stratification [52].

In this study, the incidence of PONV in the PACU was defined as early PONV, while the same incidence in the Department of Laryngology (DoL) was defined as late PONV. Overall, PONV was defined as the presence of early and/or late PONV.

### 4.5. Statistical Analysis

MS Excel 2019 and STATISTICA 13 (StatSoft, Krakow, Poland) were used for the statistical analysis. Data are presented as the mean ± standard deviation (X ± SD) or median (interquartile range). The normality of the distribution was verified by the Shapiro–Wilk test. A one-way analysis of variance (ANOVA) for multiple groups was used to examine differences between the means. For skewed distributions, either the Mann–Whitney U test or the Kruskal–Wallis test by rank was used. In addition, post hoc tests were performed to confirm differences between the groups. For nominal data, percentages were used, and pairwise comparisons between pairs of proportions with correction for multiple testing were calculated. The χ^2^ test was used to analyze the relationships between nominal variables. A *p*-value < 0.05 was considered statistically significant. The power of our sample size is about 0.73 (alpha = 0.05).

## 5. Conclusions

The current study did not show any benefit of using nociception/antinociception balance monitoring by pupillometry or the adequacy of anesthesia to guide TIVA in terms of reducing the incidence of PONV. Surprisingly, the PRD guidance for RMF infusion resulted in a fourfold higher incidence of PONV despite the sparring effect of RMF administration compared to the control group, and this result was twice as high as in the SPI group. The low overall PONV incidence of approximately 7.9%, with an impressive 3.3% in the GA group compared to the literature data, may have been due to multimodal pharmacology using agents with direct or indirect antiemetic properties. Therefore, further studies are required in this field.

## Figures and Tables

**Table 1:** Rate of PONV in patients depends on the group.

Intraoperative Data	Total *n* = 89 (100%)	GA Group *n* = 30 (33.7%)	SPI Group *n* = 31 (34.8%)	PRD Group *n* = 28 (31.5%)	*p*
Overall PONV (yes/no) (%)	7/82 7.9%	1/29 3.3%	2/29 6.5%	4/24 14.3%	0.282
	Post-Anesthesia Care Unit (PACU)				
PONV in PACU (yes/no) (%)	4/85 4.5%	0/30 0%	1/30 3.2%	3/25 10.7%	0.132
Nausea in PACU (yes/no) (%)	4/85 4.5%	0/30 0%	1/30 3.2%	3/25 10.7%	0.132
Vomiting in PACU (yes/no) (%)	4/84 4.5%	0/30 0%	1/30 3.2%	3/25 10.7%	0.132
	Department of Laryngology (DoL)				
PONV in DoL (yes/no) (%)	3/86 3.4%	1/29 3.3%	1/30 3.2%	1/27 3.6%	0.997
Nausea in DoL (yes/no) (%)	3/86 3.4%	1/29 3.3%	1/30 3.2%	1/27 3.6%	0.997
Vomiting in DoL (yes/no) (%)	2/86 2.3%	1/29 3.3%	1/30 3.2%	1/27 3.6%	0.997
Overall medication with antiemetic properties in DoL	44/45 49.4%	10/20 33.3%	19/12 61.3%	15/13 53.6%	0.080
Paracetamol in DoL	37/52 41.6%	7/23 23.3%	17/14 54.8%	13/15 46.4%	0.036
	PONV incidence both in PACU and DoL				
PONV in PACU and DoL (yes/no) (%)	0/89 0%	0/30 0%	0/31 0%	0/28 0%	-
Nausea in PACU and DoL (yes/no) (%)	0/89 0%	0/30 0%	0/31 0%	0/28 0%	-
Vomiting in PACU and DoL (yes/no) (%)	0/89 0%	0/30 0%	0/31 0%	0/28 0%	-
Number of patients with Apfel score 0 and PONV	0	0	0	0	-
Number of patients with Apfel score 1 and PONV	3	0	1	2	-
Number of patients with Apfel score 2 and PONV	2	1	0	1	-
Number of patients with Apfel score 3 and PONV	2	0	1	1	-

GA, general anesthesia; SPI, surgical pleth index; PRD, pupillary dilatation reflex; PONV, postoperative nausea and vomiting; PACU, Post-Anesthesia Care Unit; DoL, Department of Laryngology.

**Table 2:** Apfel scores in patients and their case history data regarding group allocation.

Data	Total	GA Group	SPI Group	PRD Group	*p*
	*n* = 89 (100%)	*n* = 30 (33.7%)	*n* = 31 (34.8%)	*n* = 28 (31.5%)	
Apfel (%)	26.4 ± 10.8 21.0 (18.0)	25.3 ± 9.7 21.0 (18.0)	28.2 ± 11.9 21.0 (18.0)	25.5 ± 10.9 21.0 ± (4.5)	0.542
Gender Female/Male	33/56 37.1%/62.9%	10/20 33.3%/66.7%	13/18 41.9%/58.1%	10/18 35.7%/64.3%	0.773
Motion sickness Yes/No	0/89 0%/100%	0/30 0%/100%	0/31 0%/100%	0/28 0%/100%	-
History of PONV Yes/No	4/85 4.5%/95.5%	1/29 3.3%/96.7%	2/29 6.5%/93.5%	1/27 3.6%/96.4%	-
Number of patients with Apfel score 0	8	3	3	2	0.297
Number of patients with Apfel score 1	52	18	15	19	
Number of patients with Apfel score 2	27	9	12	6	
Number of patients with Apfel score 3	2	0	1	1	
Number of patients with Apfel score 4	0	0	0	0	-

Results are presented as mean ± standard deviation and median (interquartile range) for quantitative variables and as numbers (percentages) for nominal variables. GA, general anesthesia; SPI, surgical pleth index; PRD, pupillary dilatation reflex; PONV, postoperative nausea and vomiting.

**Table 3:** Sensitivity depending on PRD calculation.

Amplitude	PRD < 5%	5% ≤ PRD < 12%	12% ≤ PRD < 20%	PRD ≥ 20%
Sensitivity	Zero	Weak	High	Very high

PRD, pupillary dilatation reflex.

**Table 4:** Detailed description of the Boezaart Bleeding Scale.

Grade	Description
0	No bleeding
1	Slight bleeding—suctioning not required
2	Slight bleeding—suctioning occasionally required
3	Slight bleeding—suctioning frequently required; bleeding threatens the surgical field a few seconds after suction removal
4	Moderate bleeding—suctioning frequently required; bleeding threatens the surgical field directly after suction removal
5	Severe bleeding—suctioning constantly required; bleeding appears faster than can be removed by suction

## Data Availability

Data are contained within the article.

## Appendix A

**Table A1:** Anthropometric data of the patients enrolled in the study.

Data		Total	GA Group	SPI Group	PRD Group	*p*
		*n* = 89 (100%)	*n* = 30 (33.7%)	*n* = 31 (34.8%)	*n* = 28 (31.5%)	
age	(years)	50.2 ± 14.6	49 ± 15.4 51 (26)	47.7 ± 13.9 46 (21)	54.1 ± 14.4 59.5 (23)	0.22
gender	Male	56 (62.9)	20 (66.7)	18 (58.1)	18 (64.3)	0.77
	Female	33 (37.1)	10 (33.3)	13 (41.9)	10 (35.7)	
height	(cm)	171.4 ± 9 171 (12)	173.5 ± 9.3 175 (9)	170.9 ± 8.7 170 (12)	169.6 ± 9.1 170 (12)	0.26
weight	(kg)	78.2 ± 14.6 79 (20)	80.3 ± 11.5 81 (18)	78.3 ± 14.7 79 (25)	75.9 ± 17.3 74 (23.5)	0.52
BMI	(kg/m^2^)	26.5 ± 3.9 26.6 (5.2)	26.8 ± 3.9 26.9 (4.4)	26.6 ± 3.6 27 (4.6)	26.1 ± 4.2 25.2 (5.2)	0.82
BMI	norm	29 (32.6)	9 (30)	7 (22.6)	13 (46.4)	0.38
	overweight	43 (48.3)	15 (50)	18 (58.1)	10 (35.7)	
	obese	17 (19.1)	6 (20)	6 (19.4)	5 (17.9)	
ASA	I	25 (29.8)	9 (31)	11 (37.9)	5 (19.2)	0.31
	II	45 (53.6)	18 (62.1)	14 (48.3)	13 (50)	0.52
	III	14 (16.7)	2 (6.9)	4 (13.8)	8 (30.8)	0.05
LM CT scale mean	LM < 12	50 (56.2)	12 (40)	19 (61.3)	19 (67.9)	0.08
	LM ≥ 12	39 (43.8)	18 (60)	12 (38.7)	9 (32.1)	
primary ESS/revision		74 (84.1)	25 (86.2)	26 (83.9)	23 (82.1)	0.91
Samter’s triad		7 (8.3)	5 (17.2)	0 (0)	2 (7.7)	0.05
asthma		17 (20.2)	10 (34.5)	4 (13.8)	3 (11.5)	0.06
arterial hypertension		31 (36.9)	10 (34.5)	10 (34.5)	11 (42.3)	0.79
coronary artery disease		7 (8.3)	2 (6.9)	1 (3.4)	4 (15.4)	0.27

GA, general anesthesia; SPI group, surgical pleth index; PRD, pupillary dilatation reflex; BBS, Boezaart Bleeding Scale; ASA, Society of Anesthesiologists; LM CT, Lund–Mackay computed tomography.

**Table A2:** Intraoperative parameters of patients depending on the group.

Intraoperative Parameters	Total	GA Group	SPI Group	PRD Group	*p*
	*n* = 89 (100%)	*n* = 30 (33.7%)	*n* = 31 (34.8%)	*n* = 28 (31.5%)	
Total intraoperative blood loss (mL)	207.5 ± 154.3 170 (200)	283.3 ± 193.5 220 (300)	165.2 ± 100.2 150 (150)	173.1 ± 128.6 135 (189)	0.04 GA vs. SPI
Length of operation (min)	74.1 ± 32.3 73 (35)	82.6 ± 33.1 85 (40)	75.8 ± 34.2 70 (39)	63.1 ± 26.7 65.5 (35)	0.05 GA vs. PRD
Total propofol consumption (mg)	666 ± 269.5 620 (340)	762.3 ± 273.2 750 (350)	665.8 ± 237.8 650 (400)	562.9 ± 269.1 520 (285)	0.008 GA vs. PRD
Max speed of propofol infusion (mcg/kg/min)	99.10 ± 41.62 86.81 (43.51)	110.40 ± 48.12 102.04 (46.90)	94.04 ± 31.70 83.33 (35.46)	92.80 ± 39.50 78.44 (45.02)	0.15
Min speed of propofol infusion (mcg/kg/min)	65.05 ± 24.25 59.52 (36)	63.27 ± 22.46 59.52 (28.33)	65.26 ± 21.54 63.64 (31.75)	66.67 ± 29.01 58.17 (44.93)	0.88
Mean speed of propofol infusion (mcg/kg/min)	80.33 ± 27.37 73.53 (35.28)	85.73 ± 26.91 83.33 (30.15)	76.14 ± 23.28 77.80 (31.59)	79.25 ± 31.62 68.56 (36.86)	0.37
Total RMF consumption (mg)	1.6 ± 1.2 1.5 (1.1)	1.7 ± 1.1 1.5 (1)	1.8 ± 0.9 1.8 (0.9)	1.3 ± 1.4 1 (0.7)	0.005 SPI vs. PRD
Max speed of RMF infusion (mcg/kg/min)	0.38 ± 0.16 0.38 (0.25)	0.42 ± 0.21 0.38 (0.25)	0.4 ± 0.12 0.38 (0.25)	0.33 ± 0.15 0.25 (0.13)	0.02 GA vs. PRD
Min speed of RMF infusion (mcg/kg/min)	0.22 ± 0.06 0.25 (0.13)	0.21 ± 0.06 0.25 (0.13)	0.25 ± 0.05 0.25 (0)	0.21 ± 0.07 0.25 (0.13)	0.03
Mean speed of RMF infusion (mcg/kg/min)	0.31 ± 0.12 0.28 (0.12)	0.32 ± 0.14 0.28 (0.16)	0.33 ± 0.09 0.34 (0.16)	0.27 ± 0.12 0.25 (0.1)	0.007 SPI vs. PRD
Max values of BBS	2.7 ± 0.7 3 (1)	2.9 ± 0.7 3 (1)	2.5 ± 0.6 3 (1)	2.6 ± 0.7 3 (1)	0.2
Min values of BBS	1.7 ± 0.5 2 (1)	1.8 ± 0.4 2 (0)	1.7 ± 0.5 2 (1)	1.6 ± 0.5 2 (1)	0.09
Mean values of BBS	2 ± 0.4 2.1 (0.3)	2.1 ± 0.5 2.2 (0.4)	1.9 ± 0.5 2 (0.5)	2 ± 0.3 2 (0.2)	0.07
Mean time of BBS > 2	12.7 ± 15.3 5 (20)	16.3 ± 16.8 15 (30)	11.8 ± 14.9 5 (25)	9.6 ± 13.7 5 (15)	0.23
Mean number of incidences of BBS > 2	1.07 ± 1.1 1 (2)	1.4 ± 1.3 1 (2)	0.9 ± 0.9 1 (1)	0.89 ± 1 1 (1.5)	0.19
Fluid challenge (mL)	1540.34 ± 381 1500 (500)	1534.48 ± 446.83 1500 (500)	1546.77 ± 372.59 1500 (500)	1539.29 ± 326.13 1500 (275)	0.93

GA, general anesthesia; SPI group, surgical pleth index; PRD, pupillary dilatation reflex; BBS, Boezaart Bleeding Scale; RMF, remifentanil.
