# Correction: Stasiowski et al. Postoperative Nausea and Vomiting Following Endoscopic Sinus Surgery Under the Guidance of Adequacy of Anesthesia or Pupillometry with Intravenous Propofol/Remifentanil. *Pharmaceuticals* 2024, *17*, 2

**DOI:** 10.3390/ph19101499

**Published:** 2026-09-22

**Authors:** Michał J. Stasiowski, Nikola Zmarzły, Beniamin Oskar Grabarek, Jakub Gąsiorek

**Affiliations:** 1 Chair and Department of Emergency Medicine, Faculty of Medical Sciences in Zabrze, Medical University of Silesia, 41-808 Katowice, Poland; gjakub93@gmail.com; 2 Department of Anaesthesiology and Intensive Care, 5th Regional Hospital, 41-200 Sosnowiec, Poland; 3 Collegium Medicum, WSB University, 41-300 Dabrowa Gornicza, Poland; nikola.zmarzly@gmail.com (N.Z.); bgrabarek7@gmail.com (B.O.G.)

## Text Correction

There was an error in the original publication [1].

A correction has been made to the Clinical Trial Registration Number.

This study was registered in the Clinical Trials Registry (SilesianMU3 NCT03389763; 9 June 2017).

The authors state that the scientific conclusions are unaffected. This correction was approved by the Academic Editor. The original publication has also been updated.
